# Drought timing and species growth phenology determine intra-annual recovery of tree height and diameter growth

**DOI:** 10.1093/aobpla/plac012

**Published:** 2022-03-18

**Authors:** Ruth van Kampen, Nicholas Fisichelli, Yong-Jiang Zhang, Jay Wason

**Affiliations:** 1 School of Forest Resources, University of Maine, Orono, ME 04469, USA; 2 Schoodic Institute at Acadia National Park, Winter Harbor, ME, USA; 3 School of Biology and Ecology, University of Maine, Orono, ME 04693, USA

**Keywords:** Diameter, Height, Intra-annual growth, Seasonal drought, Soil moisture, Water potential

## Abstract

Droughts interact with tree phenology to drive declines in growth. As climate change makes drought more likely in the Northeastern USA, it is important to understand how droughts at different times of year will lead to reduced height and diameter growth of trees. To determine how seasonal drought may reduce intra-annual growth, we implemented spring, summer or fall droughts on 288 containerized saplings of six tree species (*Acer rubrum*, *Betula papyrifera*, *Prunus serotina*, *Juniperus virginiana*, *Pinus strobus* and *Thuja occidentalis*). We tracked weekly soil moisture, leaf water potential, height, diameter and survival of all trees before, during and after each 6-week drought. We found that the tree species that conducted the majority of their height or diameter growth in the spring were most sensitive to spring droughts (*B. papyrifera* and *Pi. strobus*). *Thuja occidentalis* also experienced significantly reduced growth from the spring drought but increased growth after the drought ended and achieved total height and diameter growth similar to controls. In contrast, summer droughts halted growth in most species for the remainder of the growing season even after the drought had ended. Fall droughts never impacted growth in the current year. These fine temporal-scale measurements of height and diameter growth suggest that tree response varies among species and is dynamic at intra-annual scales. These relatively rare data on intra-annual height growth sensitivity are important for canopy recruitment of saplings in forest ecosystems. Species-specific sensitivities of intra-annual growth to drought can inform models of forest competition in a changing climate.

## Teaser

The timing of drought events can determine if, and to what extent, tree diameter growth is reduced. However, little is known about how drought timing reduces height growth. In this article in *AoB Plants*, van Kampen *et al.*found that height growth of trees was sensitive to drought. However, some trees could compensate for reduced growth during a spring drought with increased growth post-drought. Summer drought usually truncated height growth early with no compensation post-drought. These findings improve our understanding of tree drought resilience and how drought timing can change competitive dynamics between trees.

## Introduction

Drought can drive declines in tree growth (Anderegg *et al.* 2015; Babst *et al.* 2019), even in the relatively mesic forests of the Northeastern USA (NE USA) that do not typically experience drought (Clark *et al.* 2016; D’Orangeville *et al.* 2018; Vose *et al.* 2019). Although recent and projected future droughts in NE USA forests are moderate relative to more xeric regions such as parts of the Western USA (Vose *et al.* 2019), many north-eastern tree species are not well adapted to drought (Choat *et al.* 2012; Liénard *et al.* 2016; Wason *et al.* 2018). Therefore, even moderate droughts can lead to declines in growth and carbon sequestration (D’Orangeville *et al.* 2018; Kannenberg *et al.* 2019a; McGregor *et al.* 2021). In light of predictions for future warming and an increased likelihood of droughts in the NE USA (Janowiak *et al.* 2018; Vose *et al.* 2019), it is important to determine how the growth of north-eastern forest trees responds to drought.

The effects of a drought on tree growth are partly determined by the timing of the drought relative to tree growth phenology (D’Orangeville *et al.* 2018; Kannenberg *et al.* 2019a). For example, spring and early summer droughts are likely to reduce height growth for species with truncated growth phenology that conduct a majority of height growth during that time (Kozlowski 1964; Canham *et al.* 1999). Summer droughts, however, may be more likely to impact the height growth of species with continuous or episodic growth phenology that grow when conditions are favourable (Canham *et al.* 1999). In contrast to height growth, radial growth tends to occur through a longer period of the spring and summer (D’Orangeville *et al.* 2022) and therefore is likely sensitive to drought during those periods (Zhang *et al.* 2021). Importantly, however, late summer and fall droughts are less likely to impact height or diameter growth in the same year but can have lagged effects that are realized in the following year (Kannenberg *et al.* 2019b). Despite the fact that drought effects on tree growth have been generally well studied, we still know relatively little about the effects of drought timing on intra-annual tree growth, the potential for intra-annual recovery of growth following drought and how different species may respond.

Much of our understanding of drought timing effects on trees is derived from dendrochronological studies of long-term radial tree growth. Indeed, inter-annual radial growth is particularly well studied however recent developments in dendrometers have provided insight into intra-annual and diurnal patterns of radial growth for trees (Deslauriers *et al.* 2003; McMahon and Parker 2015; Zweifel *et al.* 2021). Furthermore, studies of xylem anatomy have identified that moderate drought can reduce the size of cells produced resulting in lower overall radial growth (Abe *et al.* 2003; Arend and Fromm 2007; Rossi *et al.* 2009). Although radial growth is relatively easy to measure (access at ground level and through increment boring), patterns of height growth are a major determinant of sapling recruitment as trees compete to attain favourable light conditions in forests. Despite the importance of height growth of sapling trees for recruitment to the canopy, we know relatively little about how the phenology of height growth for many species responds to drought at different times of year.

In this study, we conducted a controlled greenhouse study to test how the timing of drought (spring, summer or fall) reduced height and diameter growth for saplings of six species (*Acer rubrum*, *Betula papyrifera*, *Prunus serotina*, *Juniperus virginiana*, *Pinus strobus* and *Thuja occidentalis*; Table 1). Our objectives were to (i) describe the intra-annual growth phenology of height and diameter for saplings of these species in high temporal resolution, (ii) determine how droughts during different seasons may reduce height and diameter growth and (iii) determine the capacity for intra-annual recovery of height and diameter growth following drought stress for these six species.

**Table 1.** Study species, degree of drought tolerance (Peters *et al.* 2015), projected habitat suitability change in New England (Janowiak *et al.* 2018), leaf phenology, tree type and relative position within each species’ range found in central Maine (Burns and Honkala 1990).

Species	Drought tolerance	Projected change in habitat suitability	Leaf phenology	Tree type	Study site’s location within native range
*Acer rubrum*	Moderate	Stable	Deciduous	Angiosperm	Central
*Betula papyrifera*	Low	Decrease	Deciduous	Angiosperm	Southern
*Prunus serotina*	High	Increase	Deciduous	Angiosperm	Northern
*Juniperus virginiana*	High	Increase	Evergreen	Gymnosperm	Northern
*Pinus strobus*	Moderate	Stable	Evergreen	Gymnosperm	Central
*Thuja occidentalis*	Low	Decrease	Evergreen	Gymnosperm	Southern

## Materials and Methods

### Experimental design

**Figure 1.**
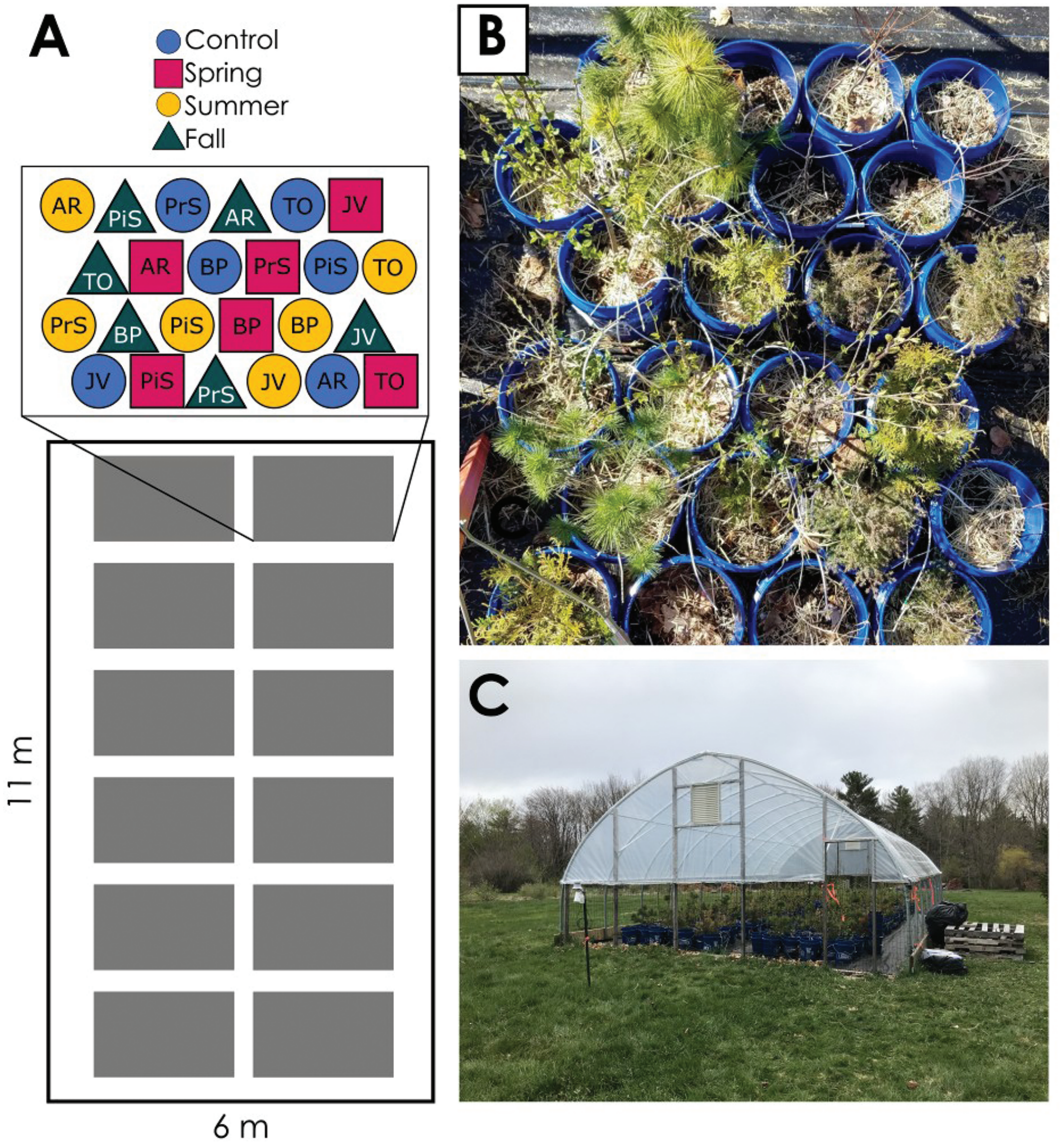
(A) Experimental layout of the high-tunnel greenhouse. The 288 saplings were arranged in 12 blocks in a randomized complete block design, with one individual of each of the six species in each of the four treatment conditions (control, spring drought, summer drought and fall drought) per block. Across all blocks, there were therefore a total of 12 replicates per species and treatment combination. Species included deciduous broad-leaved trees: *Acer rubrum* (AR), *Betula papyrifera* (BP), *Prunus serotina* (PrS) and evergreen gymnosperm trees: *Juniperus virginiana* (JV), *Pinus strobus* (PiS) and *Thuja occidentalis* (TO). (B) One experimental block of saplings in May 2020. (C) Greenhouse in May 2020 after installing the polyvinyl covering to exclude rainfall.

This experiment was conducted in a high-tunnel greenhouse in Orono, ME. In May 2019, we planted a total of 288 saplings (30–60 cm tall) of the six study species (48 saplings each of *A. rubrum*, *B. papyrifera*, *Pr. serotina*, *J. virginiana*, *Pi. strobus* and *T. occidentalis*). Each sapling was planted in a 19-L container with seven 2.5-cm drainage holes. The saplings (Cold Stream Farm, Free Soil, MI) were planted in a nursery mix of fine aged pine bark and sphagnum peat (Jolly Gardener, Poland Spring, ME) supplemented with fertilizer (5.9 g L^−1^ Osmocote 18-6-12). The saplings were arranged in a randomized complete block design with 12 experimental blocks containing 24 saplings each (four individuals per species; Fig. 1A and B). Each individual was later assigned to one of four treatments (described in the next paragraph) with 12 replicates per species and treatment combination. For the first year of growth (2019), the polyvinyl cover was left open and no treatments were applied. Each sapling was irrigated with 7.5 L of water at night three times per week during the growing season. The containerized plants were insulated with straw over the winter to avoid freezing damage to roots. In spring 2020, the polyvinyl cover was installed on the greenhouse and the sidewalls were left open to 1.2 m to limit warming (Fig. 1C). Hourly temperatures were monitored inside and outside the greenhouse with iButton loggers (iButton model DS1921G-F5# Thermochron; Maxim Integrated Products, Inc., Sunnyvale, CA) in custom radiation shields (Wason *et al.* 2017). On average, daily maximum temperatures in the greenhouse were 2.6 (±0.14), 2.6 (±0.18) and 2.3 (±0.15) °C warmer than ambient during the spring, summer and fall droughts, respectively.

In 2020, we conducted three different 6-week droughts by shutting off their irrigation. Within each experimental block (Fig. 1B), each of the four individuals of a species was randomly assigned to one of four treatments: irrigated control, spring drought (initiated 2 June), summer drought (initiated 14 July) or fall drought (initiated 25 August). In 2020, trees were irrigated with 7.5 L of water nightly (except when experiencing a drought treatment), and soil moisture (volumetric water content) was measured twice weekly in every container with a HydroSense II Handheld Soil Moisture Sensor (Campbell Scientific, Logan, UT). Weeds growing in containers were clipped weekly.

### Water status and growth measurements

To track water stress in saplings, we measured predawn leaf water potential (Ψ_PD_) in two randomly chosen blocks during the first day of the drought and weekly thereafter. During these sampling events, one leaf was collected from each of the control saplings and the saplings experiencing a drought. Leaf samples were collected at least 1 h before the first light (between 3:30 AM and 5:30 AM). For *Pi. strobus*, a 1-year-old fascicle was collected and for *J. virginiana* and *T. occidentalis*, a 2-cm sample was collected from the terminal end of a lateral branch. Samples were immediately stored in foil-lined plastic bags containing a damp paper towel and kept in a cooler until they were transported to the laboratory for measurements with a pressure chamber (Model 1000; PMS Instruments, Albany, OR). All measurements were completed within 2 h of sample collection. Midday leaf water potentials (Ψ_MD_) were measured in the third and sixth weeks of each drought to determine the maximum water stress that saplings experienced. Samples were collected between 12:30 and 2:30 PM and processed as described above to estimate Ψ_MD_.

To determine the growth phenology, we measured the height and diameter of each sapling weekly throughout the 2020 treatment period (2 June to 7 October). Height was measured along the stem from the soil surface to the tallest living bud. Diameter was calculated by averaging two perpendicular diameter measurements taken at 10 cm above the soil surface.

### Statistical analysis

Prior to initiating the drought treatments in 2020, 98 % of the *A. rubrum* and 71 % of *Pr. serotina* experienced either frost damage or browse damage to above-ground shoots. The remaining species were unaffected. Therefore, in all the following analyses, cross-species comparisons including those two species are interpreted with this potential confounding effect in mind. However, due to the regrowth of most damaged trees, treatment comparisons within species are still possible. Trees that were dead at the start of the first drought treatment were removed from all analyses (27 % of *A. rubrum*, 0 % of *B. papyrifera*, 19 % of *J. virginiana*, 13 % of *Pi. strobus*, 8 % of *Pr. serotina* and 2 % of *T. occidentalis*).

To determine how declining soil moisture was related to declining predawn and midday water potential, we fit negative exponential models to Ψ_PD_ or Ψ_MD_ as a function of soil moisture for each species. To capture differences in the initial decline in soil moisture, we also tested the rate of soil moisture decline for each species across treatments during the first 2 weeks of each drought (except for *B. papyrifera* that reached very low soil moisture within 6 days). For each sapling, we calculated a linear regression slope between soil moisture and day of drought and used an ANOVA for each species to test if the slopes differed by treatment. To approximate the duration of drought stress for each tree, we also calculated the number of days that each tree was below 10 % and 5 % soil moisture. These were estimated by fitting negative exponential curves to the decline in soil moisture for each tree and using that to determine when the tree passed 10 % and 5 % soil moisture. We then used ANOVA and Tukey’s test for each species to determine if the duration of drought stress differed by season.

To determine how drought impacted height and diameter growth, we calculated the relative height and diameter for each sapling measurement as a percent of the starting height or diameter of that sapling. We then tested those relative heights and diameters against the control trees from that same measurement with species-level Welch’s *t*-tests on each sampling day. Results are considered statistically significant for *P*-values < 0.05. Outlier height and diameter measurements (e.g. temporary large increase or decrease in height) and chronically unhealthy trees that showed little or no growth not associated with a treatment condition were removed from the analysis. Control trees were harvested for a separate experiment 2 weeks before the end of the fall drought. Therefore, the height and diameter of control trees were predicted on the final day of the fall drought using Holling type III non-linear models for each control tree (Bolker 2008). We also used these models to quantify phenology by estimating the day at which each control tree reached 90 % of its total relative height and diameter growth and tested differences among species with ANOVA and Tukey’s test. All data and figures were analysed and produced using R Version 4.0.3 (R Core Team 2021).

## Results

**Figure 2.**
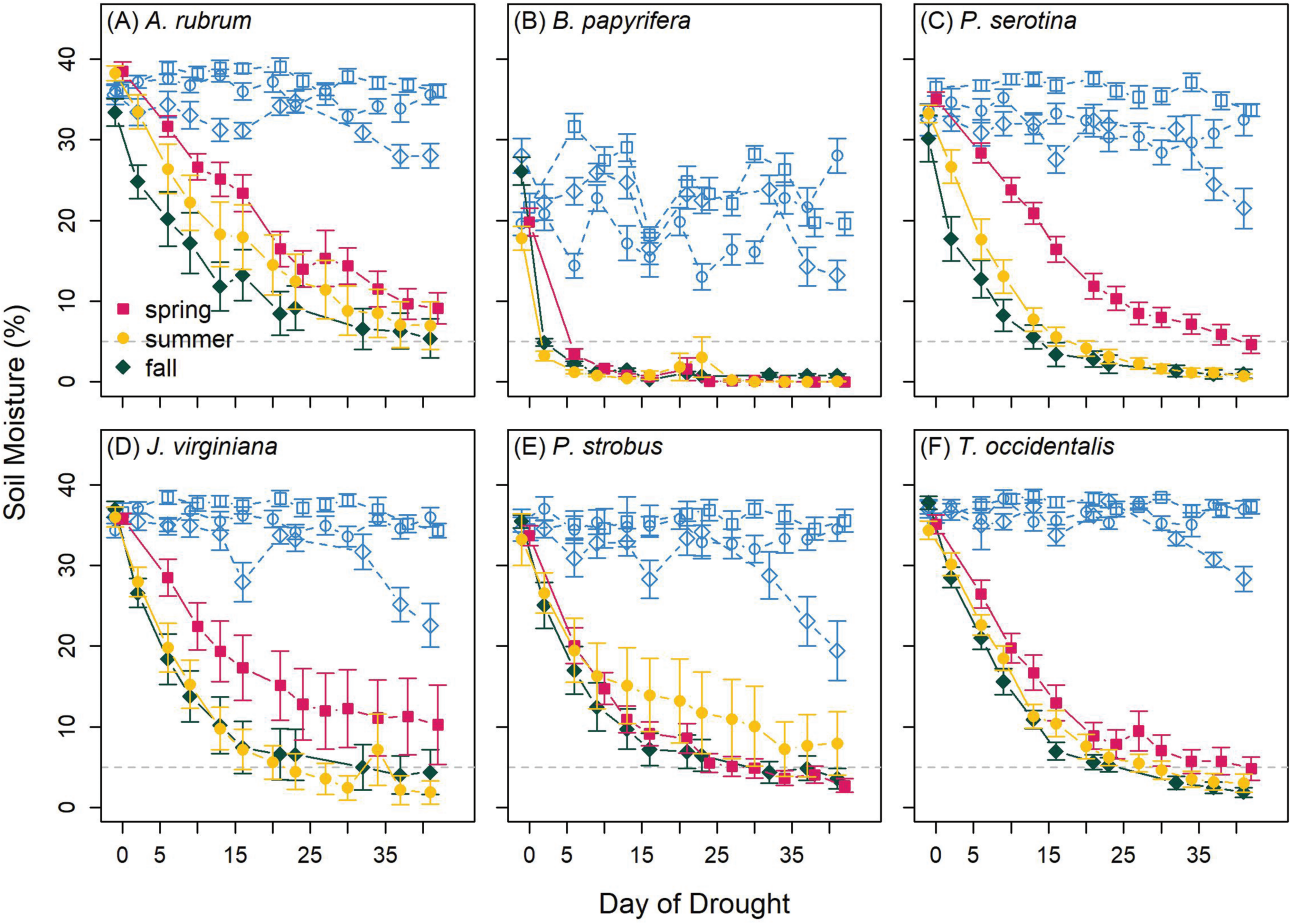
Patterns of declining soil moisture (mean ± SE) as a function of day of drought initiated during spring (filled squares), summer (filled circles), fall (filled rhombus) relative to the controls in each season (empty symbols of the corresponding shape). Each panel represents the soil moisture measurements for one of the six study species (deciduous broad-leaved trees: *Acer rubrum* (A), *Betula papyrifera* (B), *Prunus serotina* (C) and evergreen gymnosperm trees: *Juniperus virginiana* (D), *Pinus strobus* (E) and *Thuja occidentalis* (F)). A light-grey dashed line at 5 % soil moisture is on each panel to facilitate the comparison of rates of dry-down for each treatment.

We found relatively similar patterns in declining soil moisture in all seasons (Fig. 2). Although it appeared that spring droughts tended to progress more slowly than summer and fall droughts, the rate of soil moisture decline for the first 2 weeks only differed by season for *Pr. serotina* (Fig. 2C, *P* = 0.002). When comparing within-species differences in final soil moisture across drought treatments, we found that all treatments were significantly lower than controls but that the final soil moisture values for drought treatments did not differ from each other. However, we did find differences by treatment for the duration of drought stress experienced (days below 10 % or 5 % soil moisture) with *A. rubrum*, *B. papyrifera* and *Pr. serotina* each experiencing shorter periods of stress in spring than in summer or fall **[see****Supporting Information—Table S1****]**. *Betula papyrifera* experienced rapid declines in soil moisture (Fig. 2B) and was the only species that experienced drought-related mortality with low survival in the spring (8 %) and summer (8 %) droughts relative to the fall drought (100 %) and control (100 %). All other species had high survival regardless of treatment (96 % ± 6 %; mean ± SD).

**Figure 3.**
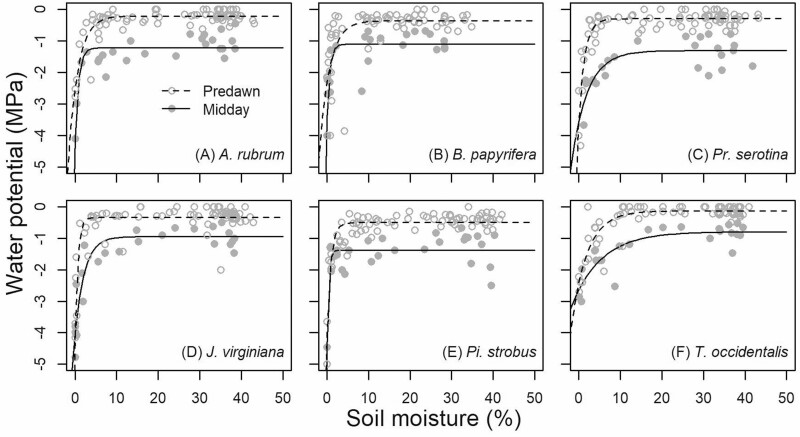
Mean Ψ_PD_ (predawn water potential; open circles) and Ψ_MD_ (midday water potential; filled circles) as a function of soil moisture. Negative exponential models (solid or dashed line) were fit for each study species (deciduous broad-leaved trees: *Acer rubrum* (A), *Betula papyrifera* (B), *Prunus serotina* (C) and evergreen gymnosperm trees: *Juniperus virginiana* (D), *Pinus strobus* (E) and *Thuja occidentalis* (F)) and for each measurement type (Ψ_PD_ or Ψ_MD_).

Leaf Ψ_PD_ and Ψ_MD_ declined for all species when the soil moisture reached values below ~5 % regardless of the season of drought (Fig. 3). At high soil moisture (asymptote of the negative exponential models), average Ψ_PD_ was between −0.13 and −0.50 MPa and average Ψ_MD_ was between −0.79 and −1.37 MPa for all species (Fig. 3 and Table 2). At high soil moisture, the average difference between Ψ_PD_ and Ψ_MD_ was 0.81 (±0.07) MPa. At low soil moisture (below 5 %), Ψ_PD_ and Ψ_MD_ converged for all species.

**Table 2.** Predicted mean (SE) Ψ_PD_ (predawn water potential) and Ψ_MD_ (midday water potential) at high soil moisture for the six study species (deciduous broad-leaved trees: *Acer rubrum*, *Betula papyrifera* and *Prunus serotina*; evergreen gymnosperm trees: *Juniperus virginiana*, *Pinus strobus* and *Thuja occidentalis*). These values were predicted as the asymptote of the negative exponential models fitted to the water potential and soil moisture data (see Fig. 3)

Species	Predicted Ψ_PD_ (MPa)	Predicted Ψ_MD_ (MPa)	Difference (MPa)
*A. rubrum*	–0.21 (0.03)	–1.22 (0.08)	1.01
*B. papyrifera*	–0.36 (0.10)	–1.10 (0.13)	0.74
*Pr. serotina*	–0.29 (0.04)	–1.31 (0.11)	1.02
*J. virginiana*	–0.33 (0.05)	–0.94 (0.13)	0.61
*Pi. strobus*	–0.50 (0.04)	–1.37 (0.09)	0.87
*T. occidentalis*	–0.13 (0.04)	–0.79 (0.12)	0.66

**Figure 4.**
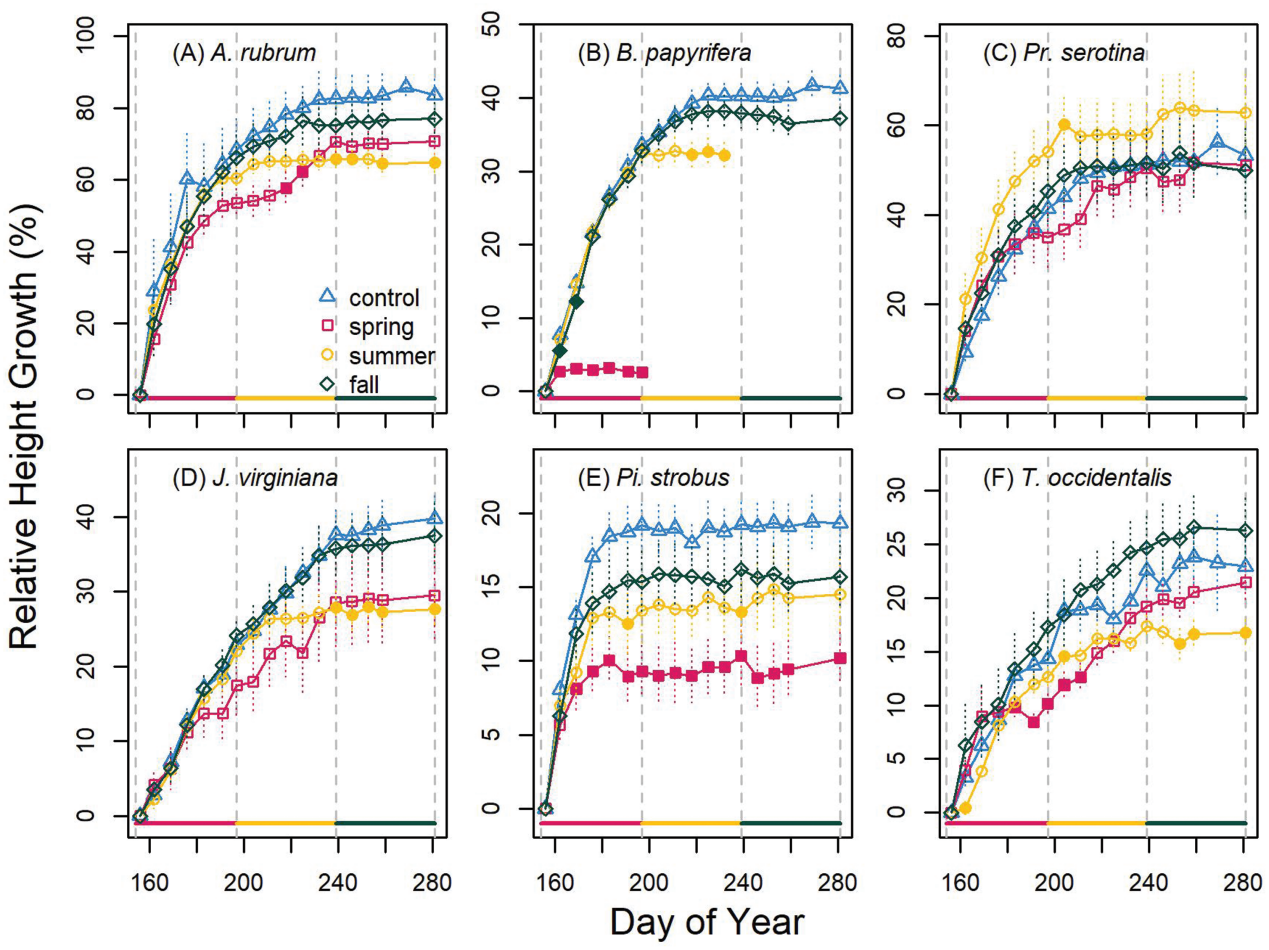
Relative height growth (per cent of initial height; mean ± SE) over the course of the treatment period (2 June to 7 October; doy 154–281) for each study species (deciduous broad-leaved trees: *Acer rubrum* (A), *Betula papyrifera* (B), *Prunus serotina* (C) and evergreen gymnosperm trees: *Juniperus virginiana* (D), *Pinus strobus* (E) and *Thuja occidentalis* (F)). Solid lines at the bottom of each panel and vertical dashed lines denote the span of each treatment (spring, summer and fall). Treatments are represented by different symbols (triangle = control, square = spring, circle = summer, rhombus = fall). If the mean relative height was significantly different from the control at *α* = 0.05, the symbol is filled.

The declines in soil moisture (Fig. 2) and associated declines in water potential (Fig. 3) coincided with reduced height and diameter growth that differed by both species and season. For control trees, *Pi. strobus* height growth finished earlier than all other species (day of year (doy) 178 ± 1.6; Fig. 4E) and *J. virginiana* height growth finished later than all other species (doy 239 ± 3.2) except *T. occidentalis* (Fig. 4B). The end of height growth for the *A. rubrum* (doy 202 ± 12.2), *B. papyrifera* (doy 208 ± 2.5), *Pr. serotina* (doy 211 ± 5.0) and *T. occidentalis* (doy 222 ± 5.1) did not significantly differ from each other. We found that spring and summer droughts resulted in reduced height growth for most species relative to controls (Fig. 4). The spring drought resulted in reduced height growth and increased mortality for *B. papyrifera* (Fig. 4B; *P* < 0.05) and permanently reduced height growth for *Pi. strobus* (Fig. 4E; *P* < 0.05). *Acer rubrum* (Fig. 4A) and *T. occidentalis* (Fig. 4F) had temporary reductions in height growth in response to the spring drought but compensated with increased growth after the drought ended resulting in no difference in final height relative to their controls (*P* > 0.05). The remaining two species (*Pr. serotina* and *J. virginiana*; Fig. 4C and D) also showed this pattern but it was not statistically significant (*P* > 0.05). Summer drought had more consistent effects on height growth than spring drought. Summer drought resulted in lower total growth for *A. rubrum* (Fig. 4A), *B. papyrifera* (Fig. 4B), *J. virginiana* (Fig. 4D) and *T. occidentalis* (Fig. 4F) and no evidence of recovery after the summer drought ended. The fall drought did not significantly limit height growth of any species (*P* > 0.05).

**Figure 5.**
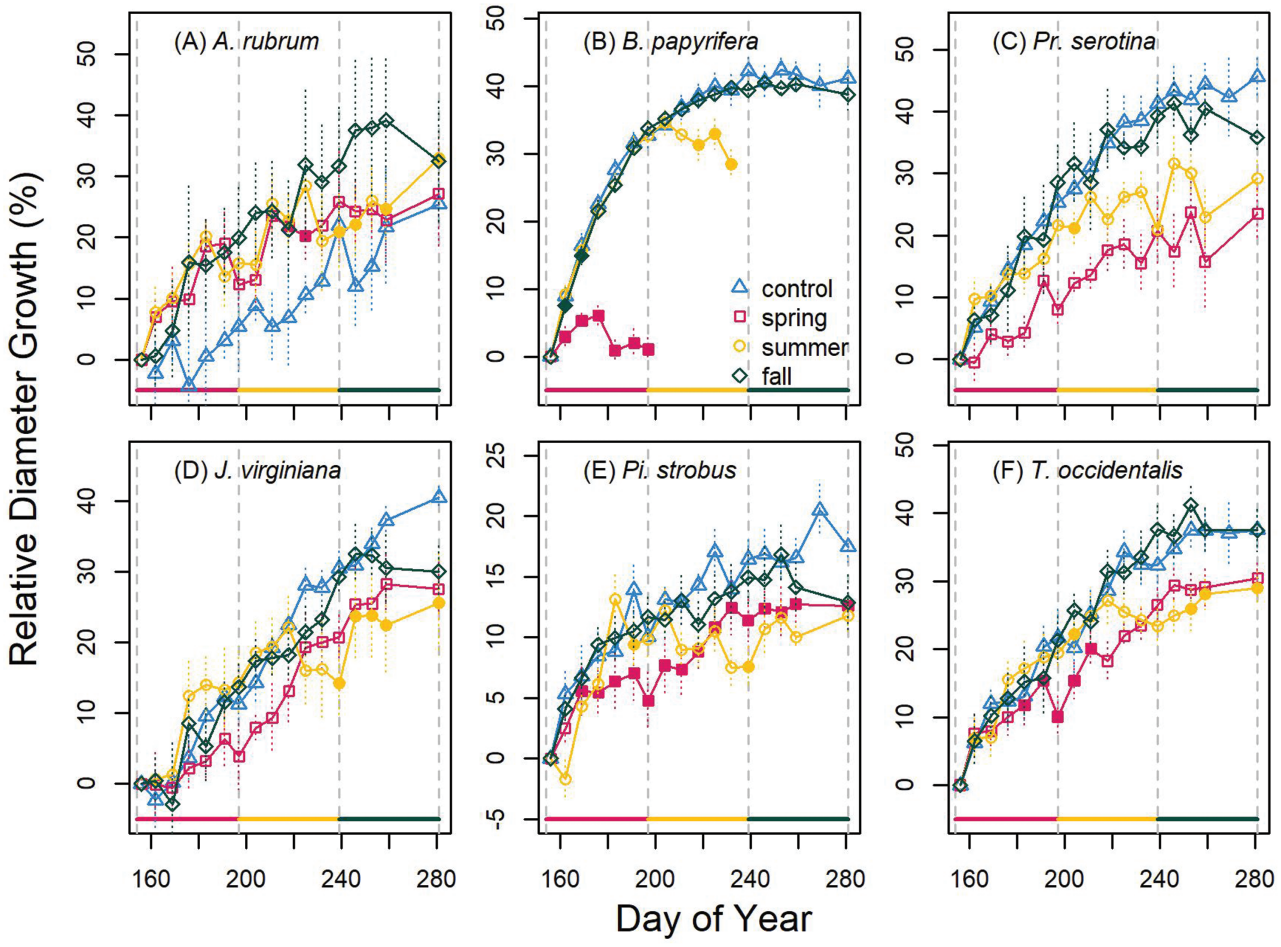
Relative diameter growth (per cent of initial diameter; mean ± SE) over the course of the treatment period (2 June to 7 October; doy 154–281) for each study species (deciduous broad-leaved trees: *Acer rubrum* (A), *Betula papyrifera* (B), *Prunus serotina* (C) and evergreen gymnosperm trees: *Juniperus virginiana* (D), *Pinus strobus* (E) and *Thuja occidentalis* (F)). Solid lines at the bottom of each panel and vertical dashed lines denote the span of each treatment (spring, summer and fall). Treatments are represented by different symbols (triangle = control, square = spring, circle = summer, rhombus = fall). If the mean relative diameter was significantly different from the control at *α* = 0.05, the symbol is filled.

Although the patterns of diameter growth are more variable than for height growth due to lower overall diameter growth relative to measurement precision, we found some consistent effects of spring and summer drought on diameter growth. Generally, control trees of *B. papyrifera* finished diameter growth earlier in the year (doy 205 ± 2.3; Fig. 5B) than all other trees except *Pi. strobus* that also finished relatively early in the year (doy 223 ± 10.6). *Pinus strobus* finished diameter growth at overlapping times with *T. occidentalis* (doy 232 ± 6.6), *Pr. serotina* (doy 235 ± 7.0) and *A. rubrum* (doy 257 ± 12.2). *Acer rubrum* and *J. virginiana* (doy 259 ± 3.5) continued diameter growth the latest into the fall. The spring drought resulted in a decline in diameter and substantial mortality for *B. papyrifera* (Fig. 5B; *P* < 0.05) and reduced diameter growth for *Pi. strobus* (Fig. 5E; *P* < 0.05) that persisted for the rest of the season. Similar to height growth, we found that *T. occidentalis* experienced temporarily reduced diameter growth from the spring drought (*P* < 0.05) but was able to compensate with increased diameter growth later in the growing season resulting in no net growth reduction (Fig. 5F; *P* < 0.05). *Acer rubrum* had the smallest initial size **[see****Supporting Information—Fig. S1****]** and frost damage (see Materials and Methods) that may have masked any patterns in diameter growth (Fig. 5A). Both *Pr. serotina* (Fig. 5C) and *J. virginiana* (Fig. 5D) exhibited a trend towards reduced diameter growth during the spring drought but this pattern was not statistically significant (*P* > 0.05). Summer drought resulted in reduced overall growth for *B. papyrifera* (and mortality; Fig. 5B), *J. virginiana* (Fig. 5D) and *T. occidentalis* (Fig. 5F). The fall drought did not significantly limit diameter growth of any species (*P* > 0.05).

## Discussion

Our study revealed how seasonal droughts could reduce the intra-annual height and diameter growth of six tree species native to the NE USA. We found that species with control trees that conducted the majority of their growth (diameter or height) in the spring were most sensitive to spring drought (*B. papyrifera* and *Pi. strobus*). The control trees of the other species (*A. rubrum*, *Pr. serotina*, *J. virginiana* and *T. occidentalis*) had extended growth phenology and continued to grow through the summer and, for some species, into the fall. These species with extended growth phenology were apparently able to compensate for spring droughts causing reduced growth by increasing growth rates in the summer and fall after the drought had ended. Interestingly, for these species with extended growth phenology, the summer drought often stopped growth for the remainder of the growing season. In contrast, fall droughts never had a significant impact on current-year growth in this study but likely would have a legacy effect that impacted growth in the following year (Kannenberg *et al.* 2019b). These detailed intra-annual data on height and diameter growth expand our understanding of how seasonal drought may impact competitive dynamics of tree saplings in a changing climate.

### Soil moisture decline and Ψ

We found a trend towards slower soil moisture declines during the spring drought relative to the summer and fall droughts for some species but this pattern was only significant for *Pr. serotina*. Slower soil moisture declines in spring were likely driven by small tree sizes and low leaf area relative to the available water (especially for frost-damaged *A. rubrum* and *Pr. serotina*). However, we still observed significant effects of the spring drought on height and diameter growth for most species (discussed further below). The summer and fall drought resulted in similar rates of soil moisture decline and duration of drought stress likely because trees had completed most of their leaf growth by mid-summer. However, despite the droughts progressing at similar rates across seasons for most species, growth phenology was completed for all species before drought stress occurred in the fall meaning that only the summer and spring drought impacted growth for some species. These findings have important implications as we consider both the changing composition of forests with different sensitivity to drought (Au *et al.* 2020) and the likelihood of drought occurring in different seasons.

Declining soil moisture resulted in lower Ψ_PD_ and Ψ_MD_ once soil moisture declined below ~5 %. At higher soil moisture (no water stress), we observed that Ψ_PD_ averaged 0.81 MPa lower than Ψ_MD_ across all species. However, once Ψ_PD_ began to decline, most species had converging Ψ_PD_ and Ψ_MD_ suggesting that they were using stomatal control to prevent midday water loss at the expense of carbon gain (Choat *et al.* 2018). Despite relatively short periods of time below 5 % soil moisture and at low Ψ_PD_ and Ψ_MD_, we still observed consistent declines in height and diameter growth. These growth declines from a moderate drought are applicable to our region where tree-ring studies sometimes find that tree growth is sensitive to precipitation despite the common assumption that these forests are not moisture-limited (D’Orangeville *et al.* 2018; Teets *et al.* 2018). Notably, very few of the trees in this experiment reached Ψ_MD_ below published values for the water potential inducing 50 % loss of hydraulic conductivity (p50) for these species (Fig. 3; p50: *A. rubrum* −2.0 to −3.3 MPa, *B. papyrifera* −2.3 MPa, *Pr. serotina* −4.3 MPa, *J. virginiana* −5.8 MPa, *Pi. strobus* −5.3 MPa and *T. occidentalis* −3.6 MPa; Tyree and Dixon 1986; Sperry *et al.* 1994; Sperry and Hacke 2004; Maherali *et al.* 2006; Willson *et al.* 2008; Hoffmann *et al.* 2011; Choat *et al.* 2012; Wason *et al.* 2019; Asbjornsen *et al.* 2021). The relatively high water potentials experienced in this study further suggest the declines in growth were not driven by hydraulic failure from an extreme drought that would be more likely to trigger embolism spread and mortality (Hammond *et al.* 2019).

### Seasonal growth patterns

The effect of drought timing was clearly related to the growth phenology observed in the treatment and well-watered control plants. Growth phenology fell generally into two groups. One group consisted of two species that conducted most of their height (*Pi. strobus*) or diameter (*B. papyrifera*) growth in spring. The second group consisted of the remaining four species (*A. rubrum*, *Pr. serotina*, *J. virginiana* and *T. occidentalis*) that conducted height growth in spring into late summer (*J. virginiana* ending height growth the latest) and diameter growth from spring into the fall (*A. rubrum* and *J. virginiana* ending diameter growth the latest). We generally found that droughts reduced growth rates most when trees would normally experience rapid growth. We also found that if these moderate droughts ended early enough in the species-typical growth pattern (spring drought for most species), they could compensate with increased growth rates after the drought. Intra-annual compensatory growth following release from drought stress has been documented in seedlings of other species (Seidel *et al.* 2019). Although we did not measure the physiology driving this compensation, it has been hypothesized to be driven by reduced phloem transport during drought leading to a build-up of non-structural carbohydrates that are rapidly allocated to growth following release from drought stress (Seidel *et al.* 2019). These results demonstrating intra-annual resilience of tree growth to drought illuminate response patterns not captured at inter-annual scales (Arend *et al.* 2011; Bottero *et al.* 2017). However, the intra-annual resilience observed in some of our species (e.g. *T. occidentalis*) may still result in lagged effects the following year due to the shortened period of potential carbon gain caused by the drought. Indeed, our results support the general notion that evergreen species tend to exhibit smaller lagged effects of drought in subsequent years because they have a longer growing season for carbon assimilation (Kannenberg *et al.* 2019a).

In the light-limited forests of the NE USA, height growth is one of the most important factors for species success early in stand development. Our data reveal the important limitations that drought can have on intra- and inter-annual height growth that may change competitive dynamics. For species with truncated growth phenology that conduct a majority of height growth in spring (Canham *et al.* 1999), our results suggest a moderate spring drought caused early cessation of height growth with no recovery. In contrast, for species with continuous growth phenology (Canham *et al.* 1999), our results suggest that they could often recover from spring drought although growth was still halted by a summer drought. These results appear to overwhelm the general pattern of shoot growth of north-eastern temperate trees being determined by temperature and moisture conditions during bud formation in the prior year (Kozlowski 1964). These growth reductions do not necessarily result from less carbon gain that year. Instead, the carbon and nutrients taken that year may be allocated to storage (Schott *et al.* 2013) and used for growth in the following year. Therefore, in a forest setting, trees with reduced height growth from drought may be partially shaded by competition at the start of the next season and have a slight competitive disadvantage despite potentially having prior year resources to allocate to new growth. These results underscore the need for future studies to continue to explore the lagged effects of seasonal drought in the years after the drought (Itter *et al.* 2019; Kannenberg *et al.* 2019b), especially on the important metric of height growth for sapling trees (Pregitzer and Palik 1993; Gutsell and Johnson 2002). Furthermore, we need to expand beyond this approach of individually potted trees to drought responses of communities of competing plants (Kerr *et al.* 2021) to improve our understanding of the connections between physiological mechanisms driving reduced growth and impacts on forest dynamics (McDowell *et al.* 2020).

Radial growth, in contrast to height growth, has been much more studied relative to drought through dendrochronological, dendrometer and xylem anatomy studies. However, although latewood and earlywood sensitivity to climate (Michelot *et al.* 2012; D’Orangeville *et al.* 2013; Pompa-García *et al.* 2021) as well as xylem anatomical features (Rossi *et al.* 2009) generally reflect the season in which they form, the most common studied feature is total ring-width sensitivity to climate. Our study provides further justification for the value of intra-annual measurements, e.g. using dendrometer bands (Ruehr *et al.* 2016; D’Orangeville *et al.* 2022), that can identify species sensitivity to drought (Etzold *et al.* 2022) and potential for intra-annual recovery. For example, we observed reduced diameter growth rates in *T. occidentalis* during a spring drought that were compensated for by increased growth in summer and fall. The resulting total radial growth did not differ between *T. occidentalis* saplings that experienced spring drought and the controls. These new results on intra-annual sensitivity suggest that indeed *T. occidentalis* (and potentially other tree species not included in this study) are sensitive to moderate drought but, at least within one growing season, they are able to compensate for this reduction with plasticity in growth phenology.

Although the results of drought experiments on potted plants are strongly linked to the size of the tree, its overall water use and the available volume of water at the start of the drought, we are able to make some conclusions across the species in this study about resistance and resilience of growth to drought. It has been suggested that shade-intolerant species are more likely to exhibit continuous growth during favourable conditions, whereas shade-tolerant species exhibit truncated growth (Marks 1975; Canham *et al.* 1999; Gaucher *et al.* 2005). In our study, we did not find this pattern. For example, within the evergreen gymnosperms we found that *Pi. strobus* (moderately shade-tolerant) exhibited strongly truncated height growth, whereas *J. virginiana* (also moderately shade-tolerant) and *T. occidentalis* (very shade-tolerant) exhibited continuous height growth. Therefore, our results suggest that shade tolerance is not an adequate proxy for growth phenology (Marks 1975; Gaucher *et al.* 2005).

## Conclusions

We found that drought timing and species growth phenology were the most important factors determining how intra-annual tree height and diameter growth responded to drought. Generally, trees that conduct most height or diameter growth during a short period of time are very sensitive to drought during that period and have limited ability to compensate later in the growing season. In contrast, species that have extended growth phenology were better able to take advantage of post-drought conditions to compensate for growth reductions during early-season droughts. As climate continues to warm and growing seasons lengthen, species that are able to extend their growth phenology (Geng *et al.* 2020; Montgomery *et al.* 2020) may be in a better position to recover from early-season droughts with later-season compensatory growth. These results can lead to better understandings of how climate change may impact early competitive dynamics of trees that can inform forest growth, carbon uptake and carbon storage.

## Supporting Information

The following additional information is available in the online version of this article—

Table S1. Duration of drought stress for each tree species and drought.

Figure S1. Average initial height of trees by species.

plac012_suppl_Supplementary_Material

## Data Availability

The data from this study are available at: doi:10.5061/dryad.b8gtht7f7

## Notes

**Plants, Ecosystems & Climate. Chief Editor**: Mary Heskel
