# Supplementary material for: Drought timing and species growth phenology determine intra-annual recovery of tree height and diameter growth
Source: AoB Plants. 2022 Mar 18;14(3):plac012. doi: 10.1093/aobpla/plac012 (PMC9089829; doi:10.1093/aobpla/plac012)
Supplement: plac012_suppl_Supplementary_Material [file plac012_suppl_supplementary_material.pdf]

Supporting Information

Title: Drought timing and species growth phenology determine intra-annual recovery of tree height and diameter growth

Authors: Ruth van Kampen, Nicholas Fisichelli, Yong-Jiang Zhang, and Jay Wason

Table S1. Duration of drought stress (days) for six tree species in the spring, summer, and fall treatment conditions for the six study species: *Acer rubrum*, *Betula papyrifera*, *Prunus serotine*, *Juniperus virginiana*, *Pinus strobus*, and *Thuja occidentalis*. Duration of drought stress was calculated by fitting negative exponential models to the relationship between day of drought and soil moisture for each tree and averaging the number of days below 10% and 5% soil moisture for each species and treatment combination. Values represent means with standard errors in parentheses. Superscript letters denote significant differences (Tukey's HSD, alpha = 0.05) within a species and soil moisture threshold.

		Days < 10%			Days < 5%	
Species	Spring	Summer	Fall	Spring	Summer	Fall
A. rubrum	8.0 (3.4) [a]	22.8 (4.3) [b]	22.0 (4.5) [b]	3.8 (2.0) [a]	16.6 (4.3) [a]	12.8 (5.2) [a]
B. papyrifera	39.6 (0.4) [a]	41.8 (0.1) [b]	41.3 (0.1) [b]	37.3 (0.6) [a]	40.7 (0.3) [b]	40.0 (0.2) [b]
Pr. serotina	15.3 (2.3) [a]	30.1 (1.8) [b]	34.1 (2.0) [b]	7.0 (2.0) [a]	23.6 (2.6) [b]	27.5 (3.6) [b]
J. virginiana	24.9 (4.0) [a]	26.7 (3.8) [a]	26.4 (4.5) [a]	19.0 (4.6) [a]	18.7 (4.2) [a]	22.3 (4.0) [a]
Pi. strobus	25.0 (3.0) [a]	28.8 (4.2) [a]	27.2 (4.3) [a]	16.3 (3.3) [a]	21.9 (5.0) [a]	20.7 (4.2) [a]
T. occidentalis	18.5 (3.3) [a]	23.8 (2.4) [a]	27.2 (1.5) [a]	9.9 (3.0) [a]	15.0 (2.5) [a]	17.5 (2.9) [a]

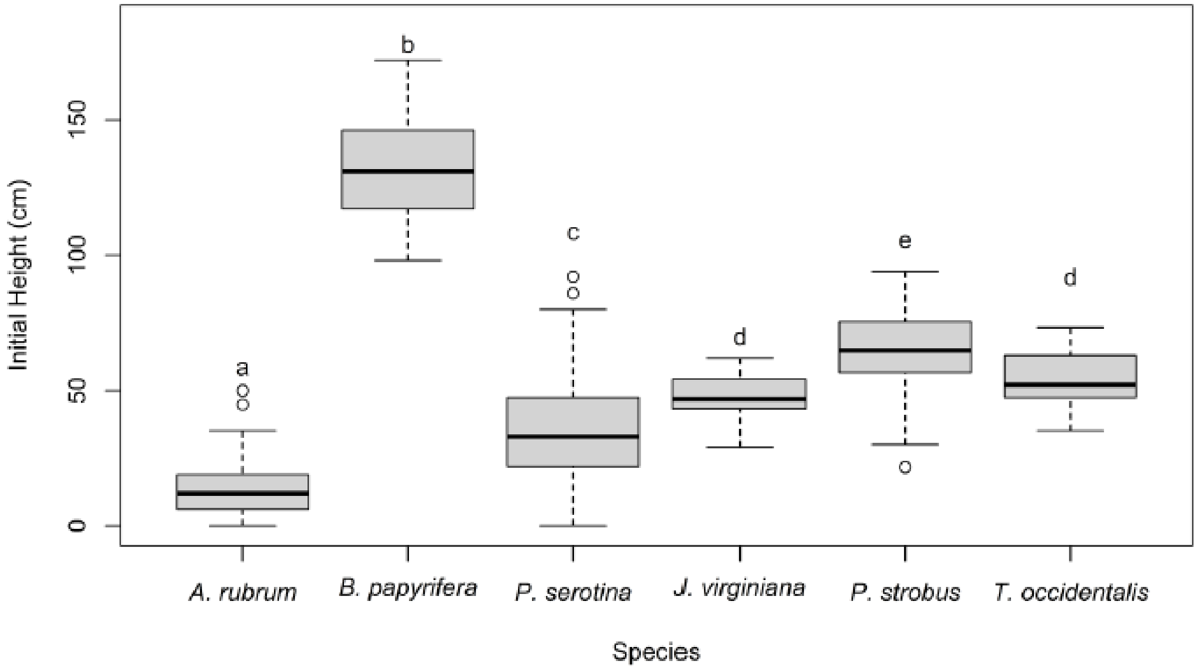

Figure S1. Boxplots of initial height in May 2020 (cm) for each of the study species (deciduous broad-leaved trees: *Acer rubrum*, *Betula papyrifera*, and *Prunus serotina*; and evergreen gymnosperm trees: *Juniperus virginiana*, *Pinus strobus*, and *Thuja occidentalis*). Differences between species are denoted with letters (ANOVA with Tukey's HSD, alpha = 0.05).
